# Supplementary material for: PMF-GRN: a variational inference approach to single-cell gene regulatory network inference using probabilistic matrix factorization
Source: Genome Biol. 2024 Apr 8;25:88. doi: 10.1186/s13059-024-03226-6 (PMC11003171; doi:10.1186/s13059-024-03226-6)
Supplement: Supplementary file 1 — Additional file 1. Additional tables [62–115]. [file 13059_2024_3226_MOESM1_ESM.pdf]

Additional File 1

Method	*B. subtilis* Cross-Validation Dataset B1		
	Regular	No Prior	Shuffled
No Normalization	0.0509 ± 0.0273	0.0048 ± 0.0003	0.0050 ± 0.0028
Min-Max Scaling	0.1931 ± 0.0171	0.0042 ± 0.0003	0.0042 ± 0.0018

Table S1: AUPRCs achieved by PMF-GRN on *B. subtilis* B1 dataset. Results are reported as the mean AUPRC across five 'cross-validation' splits ± standard deviation

Method	*B. subtilis* Cross-Validation Dataset B2		
	Regular	No Prior	Shuffled
No Normalization	0.2508 ± 0.0232	0.0062 ± 0.0006	0.0052 ± 0.0004
Min-Max Scaling	0.2886 ± 0.0312	0.0048 ± 0.0008	0.0038 ± 0.0005

Table S2: AUPRCs achieved by PMF-GRN on *B. subtilis* B2 dataset. Results are reported as the mean AUPRC across five 'cross-validation' splits ± standard deviation

Method	Input	Output	Methodology	Pipeline
PMF-GRN	(1) one or more scRNA-seq datasets<br>(2) priors constructed from genomic data such as ATAC-seq or ChIP-seq and TF motifs or literature database derived priors	(1) individual (and) combined GRN<br>(2) TFA	probabilistic matrix factorization, variational inference	1) hyperparameter search using 80-20 split of input prior<br>(2) GRN inference with optimal model parameters<br>(3) evaluate GRNs with AUPRC, MCC and F1 scores and uncertainty calibration
SCENIC	(1) scRNA-seq<br>(2) a list of TFs ranking databases for motif analysis	(1) GRN: loom file containing regulons (interactions between TFs and their target genes)<br>(2) TFA: biological activity of regulons in a given cell	tree based regression models, gradient boosting machine regression, motif enrichment analysis, and gaussian mixture models for regulon activity binarization	(1-4) preprocessing steps<br>(5) network inference<br>(6) module generation<br>(7) motif enrichment and TF regulon prediction<br>(8) cellular enrichment<br>(9) optional binarization of cellular regulon activity<br>(10) clustering of cells based on regulon activity
CellOracle	(1) ATAC-seq + TF motifs<br>(2) scRNA-seq	(1) GRN<br>(2) cell-state transition vectors after gene perturbation	Bayesian Ridge regression	(1) base GRN construction with scATAC-seq or promoter databases<br>(2) scRNA-seq preprocessing<br>(3) context dependent GRN inference<br>(4) network analysis<br>(5) simulation of cell identity after TF perturbation<br>(6) calculation of pseudotime gradient for perturbation scores
AMuSR	(1) two or more scRNA-seq datasets<br>(2) one or more priors constructed from genomic data and TF motifs or literature database derived priors	(1) individual and combined GRNs<br>(2) TFA	Regularized linear regression	(1) estimating TFA<br>(2) learning regression parameters<br>(3) model selection with bayesian information criterion<br>(4) evaluate GRNs with AUPRC, MCC, F1 scores
BBSR	(1) one or more scRNA-seq datasets<br>(2) one or more priors constructed from genomic data and TF motifs or literature database derived priors	(1) individual (and) combined GRN<br>(2) TFA	Bayesian best subset regression	(1) estimate TFA<br>(2) learn regression parameters<br>(3) model selection<br>(4) evaluate GRNs with AUPRC, MCC, F1 scores
StARS	(1) one or more scRNA-seq datasets<br>(2) one or more priors constructed from genomic data and TF motifs or literature database derived priors	(1) individual (and) combined GRN<br>(2) TFA	Least absolute shrinkage and selection operator combined with the Stability Approach to Regularization Selection	(1) estimate TFA<br>(2) learn regression parameters<br>(3) model selection<br>(4) evaluate GRNs with AUPRC, MCC, F1 scores

Table S3: GRN inference method comparison table. Table includes input data, output, methodology and pipeline organization for each of the six GRN inference methods discussed in this work.

Method	Prior Information		
	Regular	None	Shuffled
PMF-GRN	0.375	0.014	0.023
AmUSR	0.223	0.024	0.019
BBSR	0.402	0.022	0.018
StARS	0.186	0.028	0.017
SCENIC	0.014	0.014	0.014
CellOracle	0.383	N/A	0.013

Table S4: AUPRCs achieved by PMF-GRN, the Inferelator algorithms (AMuSR, BBSR, and StARS), Scenic and CellOracle on *S. cerevisiae* datasets.

Method	Cross Validation Split				
	Split 1	Split 2	Split 3	Split 4	Split 5
PMF-GRN	0.114	0.096	0.086	0.1342	0.118
BBSR	0.112	0.128	0.161	0.171	0.139
StARS	0.109	0.137	0.154	0.195	0.151
SCENIC	0.020	0.021	0.018	0.025	0.021
CellOracle	0.034	0.042	0.034	0.043	0.034

Table S5: AUPRCs achieved by PMF-GRN, the Inferelator algorithms (BBSR, and StARS), Scenic and CellOracle on *S. cerevisiae* datasets using the gold standard for 5-fold cross validation.

Method	Noise Added			
	No Noise	100% Noise	250% Noise	500% Noise
PMF-GRN	0.343	0.280	0.198	0.149
BBSR	0.264	0.208	0.186	0.174
StARS	0.136	0.125	0.114	0.118
SCENIC	0.075	0.068	0.059	0.055
CellOracle	0.417	0.306	0.226	0.175

Table S6: AUPRCs achieved by PMF-GRN, the Inferelator algorithms (BBSR, and StARS), Scenic and CellOracle on *S. cerevisiae* datasets using increasing amounts of noise added to the prior-knowledge data.

Method	Intersection over Union (IoU)
PMF-GRN	15.69%
BBSR	14.56%
AMuSR	12.46%
StARS	11.78%
SCENIC	3.17%
CellOracle	30.28%

Table S7: Intersection over Union (IoU) scores achieved by PMF-GRN, the Inferelator algorithms (AMuSR, BBSR, and StARS), Scenic and CellOracle for GRNs learned on individual *S. cerevisiae* datasets.

Expression size (%)	Sample Number				
	1	2	3	4	5
80%	0.2838	0.2947	0.3395	0.3325	0.2774
60%	0.2382	0.3295	0.3252	0.2775	0.3296
40%	0.3157	0.2723	0.3599	0.2858	0.2939
20%	0.2995	0.3503	0.3229	0.2868	0.3335

Table S8: AUPRCs achieved by PMF-GRN across 4 different downsample sizes (80%, 60%, 40%, and 20%), across 5 samples for each downsample size.

Cross Validation Split	Dataset					
	1	2	3	4	5	Full GRN
80% train - 20% val	0.0460	0.0400	0.0463	0.0361	0.0408	0.3337
60% train - 40% val	0.0479	0.0378	0.0463	0.0417	0.0437	0.3337
40% train - 60% val	0.0459	0.0397	0.0375	0.0347	0.0381	0.2909
20% train - 80% val	0.0401	0.0434	0.0403	0.0427	0.0239	0.2882

Table S9: AUPRCs achieved by PMF-GRN across 4 different cross-validation splits. 5 hyperparameters searches were performed for each cross-validation split. Full GRN was inferred using the hyperparameters for the best overall AUPRC per cross-validation split.

TF family	TF	Gene	Citation
STAT	STAT1	IRF1	[62]
		IRF9	[63]
		KDM6B	[64]
		SPAG9	[65]
	STAT3	IL24	[66], [67]
		IRF9	[68]
		ADAM12	[69]
		CRTC3	[70]
		CXCL2	[71]
		GOLPH3	[72]
		IQGAP1	[73]
		NEDD4L	[74]
		NRL	[75]
		PBX1	[76]
		SLC9A8	[77]
		YAP1	[78]
		IRF9	[68]
		ANXA4	[79]
	STAT5	AUTS2	[80]
		EPAS1	[47]
GATA	GATA1	ATP2B4	[81]
		FOXO3	[82]
		GATA2	[83]
		LAPTM5	[84]
		LYL1	[85]
		PBX1	[86]
	GATA2	CUX1	[87]
	GATA3	ABCC3	[88]
		EGFR	[89]
		ETS2	[90]
		FOS	[91]
		FOSL2	[92]
		IQGAP1	[93]
		KLF6	[94]
		RUNX2	[95]
		ZNF462	[96]
	GATA4	EPAS1	[97]
		HNF4A	[98]
		IL1R1	[99]
		YAP1	[100]
IRF	IRF1	B2M	[46]
		BTN3A1	[47]
	IRF2	B2M	[48]
	IRF3	GPR108	[49]
		RNF5	[50]
		TRAF2	[51]
SMAD	SMAD1	CHD7	[101]
	SMAD3	LAPTM5	[102]
		LIN28A	[103]
		MSL2	[104]
		TRIB3	[105]
	SMAD4	CEBPB	[106]
		CXXC5	[107]
		GDF15	[108]
		HNF4	[109]
		ROCK2	[110]
		ULK1	[111]
		WWOX	[112]
EGR	EGR1	JUND	[113]
		NEDD4L	[114]
		NME1	[115]

Table S10: Literature supported interactions for PBMC GRNs for STAT, GATA, IRF, SMAD and EGR immune TF families
