# Supplementary material for: PMF-GRN: a variational inference approach to single-cell gene regulatory network inference using probabilistic matrix factorization
Source: Genome Biol. 2024 Apr 8;25:88. doi: 10.1186/s13059-024-03226-6 (PMC11003171; doi:10.1186/s13059-024-03226-6)
Supplement: Supplementary file 2 — Additional file 2. Additional figures. [file 13059_2024_3226_MOESM2_ESM.pdf]

Additional File 2

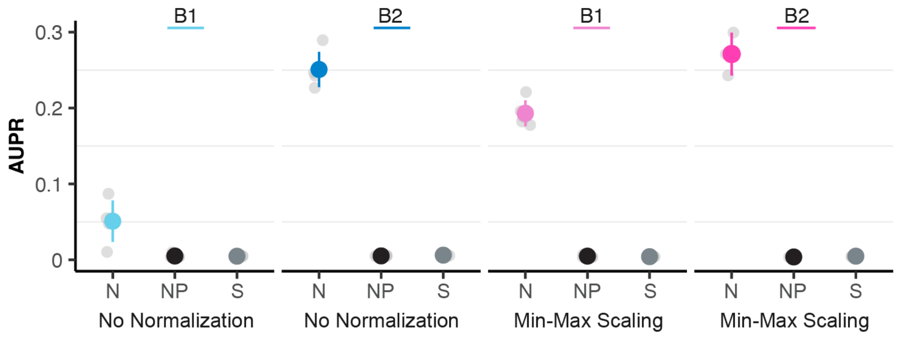

Figure S1: Results for GRNs learned in *B. subtilis* datasets B1 (GSE27219) and B2 (GSE67023) comparing "No Normalization" to "Min-Max Scaling". Colored dots represent the normal (N) GRN, with the line indicating the mean of the cross-validation experiments ± standard deviation. Negative controls are demonstrated by black dots for GRNs inferred with No Prior (NP) and grey dots for Shuffled Prior (S).

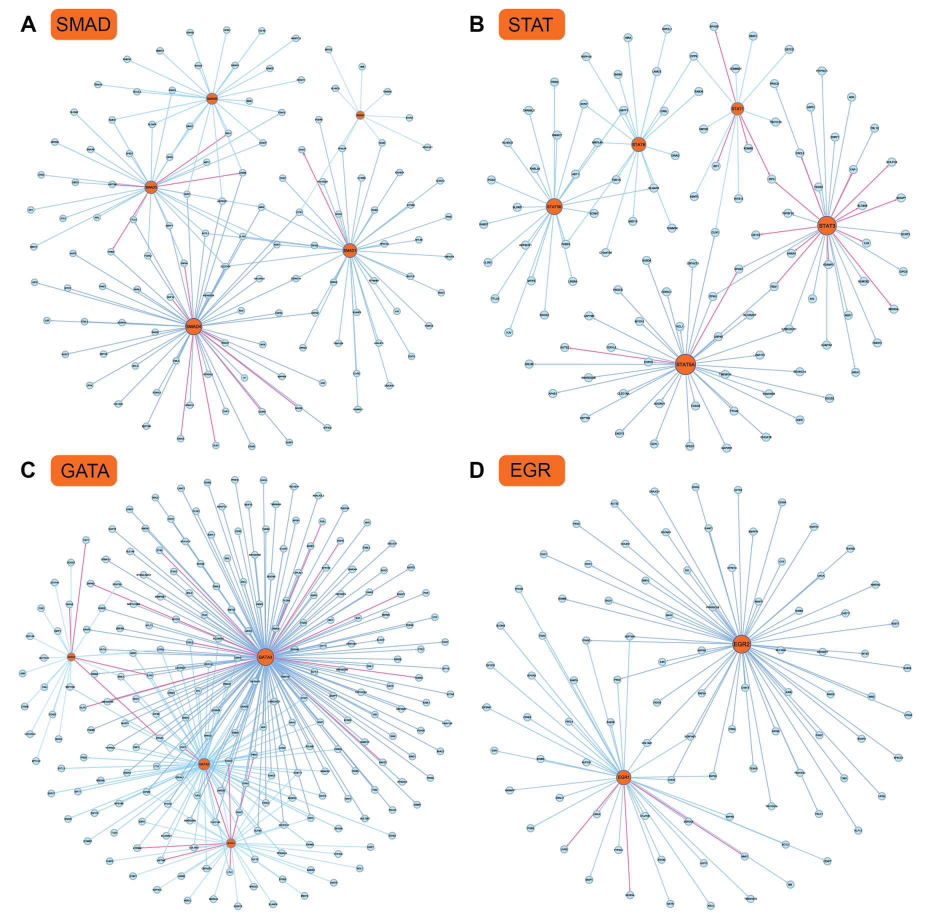

Figure S2: PBMC GRN graphs for the family of TFs belonging to (**A**) SMAD, (**B**) STAT, (**C**) GATA, and (**D**) EGR. TFs for each graph are represented by orange nodes, while target genes are blue nodes. Pink regulatory edges represent interactions with supporting literature. Color scale for blue regulatory edges are scaled from light blue (less out-degree regulation) to darker blue (more out-degree regulation).

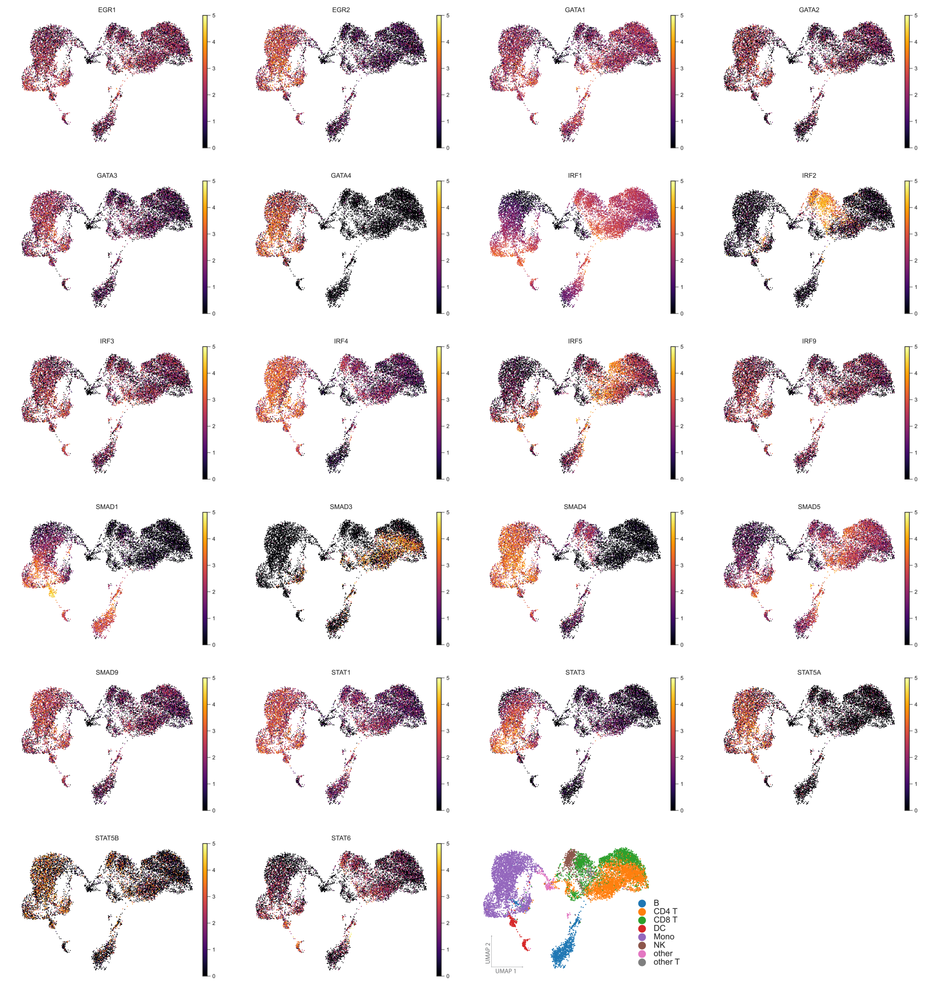

Figure S3: UMAP of predicted PBMC TFA. Each UMAP highlights the specific location of activity for each TF considered from the immune PBMC TFs. The final UMAP serves as a reference to TFA annotated by cell-type.
