# Supplementary material for: PMF-GRN: a variational inference approach to single-cell gene regulatory network inference using probabilistic matrix factorization
Source: Genome Biol. 2024 Apr 8;25:88. doi: 10.1186/s13059-024-03226-6 (PMC11003171; doi:10.1186/s13059-024-03226-6)
Supplement: Supplementary file 3 — Additional file 3. Additional experiments [116–120]. [file 13059_2024_3226_MOESM3_ESM.pdf]

Additional File 3

PMF-GRN Recovers True Interactions in Prokaryotes as Evaluated by Cross-Validation

To demonstrate GRN inference on a forth additional dataset, we carry out experiments using two microarray datasets for the prokaryote *Bacillus Subtilis* (B1 - GSE27219 [116] and B2 - GSE67023 [117]). Although PMF-GRN is not primarily designed to learn GRNs from microarray data, we show that it is still possible to learn informative GRNs with this data. For our *B. subtilis* experiments, we have access to prior-knowledge derived from the subtiwiki database [118, 119, 120]. Here, we implement a 5 fold cross-validation approach by using five random splits of the subtiwiki database-derived information, where 80% is used as prior knowledge and 20% is used as the gold standard for evaluation.

The two *B. subtilis* datasets were previously normalized after data collection as part of standard microarray processing. However, each dataset was normalized using different approaches (described in Methods). For B1, the expression data underwent no further normalization and was simply converted to integers to simulate single-cell-like data. For B2, the expression data was re-scaled and then converted to integers, in order to contain only positive integers resembling single-cell-like data. The results from our experiments are shown in Figure S1, and the numbers used to create this figure are given in Table S1 and S2. Using five repeats of cross-validation, we show the performance of GRNs inferred for the two *B. subtilis* datasets (B1 and B2). We remark that the difference in performance between B1 and B2 is likely a result of the chosen microarray processing normalization. To further support this claim, we demonstrate GRN performance after re-scaling the data with min-max scaling (Figure S1).

'No Prior' and 'Shuffled' results are also shown in Figure S1 by black and gray dots respectively. Here, we are able to demonstrate that for B1 and B2, each GRN yields a better performance as compared to negative controls.
