# Supplementary material for: PMF-GRN: a variational inference approach to single-cell gene regulatory network inference using probabilistic matrix factorization
Source: Genome Biol. 2024 Apr 8;25:88. doi: 10.1186/s13059-024-03226-6 (PMC11003171; doi:10.1186/s13059-024-03226-6)
Supplement: Supplementary file 4 — Additional file 4. Additional methods, including data generation and curation [121–123]. [file 13059_2024_3226_MOESM4_ESM.pdf]

Additional File 4

TF Target Gene Connectivity Matrix Generation

Saccharomyces cerevisiae

Datasets were obtained from [31] without further modification.

Peripheral Blood Mononuclear Cells

Datasets were obtained from [43] without further modification.

BEELINE

Datasets were obtained from [37]. TF-target gene matrices were constructed by creating cross-tab matrices from the RefGRN. 50% of this matrix was used as prior-knowledge, the remaining 50% was used as a gold standard for evaluation.

Bacillus subtilis

A prior-known TF-target gene interactions matrix was obtained from the Subtiwiki database [121] from "regulations" (downloaded 07/21/22). Using the columns "regulator locus" and "gene locus" a cross-tab integer matrix was created, where 1 represents the existence of an interaction and 0 represents no interaction. This matrix was randomly split 5 times in 80%-20% proportions along the gene axis to generate independent prior-known information and gold standard matrices.

Peripheral Blood Mononuclear Cells

For GRN inference in PBMC, we perform a hyperparameter search using a 5 fold cross-validation split of the prior-knowledge matrix. Here, 80% of the prior-knowledge is used for training, while the remaining 20% is used for validation. For PBMC, we do not have access to a gold standard network. For this reason, to evaluate our inferred GRNs, we select the network for each of the 5 cross-validation splits that achieves the best training hyperparameters. We then take the intersection of each of these 5 optimal networks, and filter the predicted interactions by those obtaining predicted means (probability) of > 0.90 across every split.

For TFA inference, we select the best overall hyperparameters from the 5 fold cross-validation hyperparemeter search to infer a single network. We do this in order to obtain a single TFA matrix where the entries of this matrix are not affected by averaging across multiple datasets. All predicted TFA values are non-zero by nature of matrix factorization. We thus apply an $l1$ regularization on the matrix with $\lambda = 1$ to push low scoring activity values to 0.

UMAP projections were performed on this regularized TFA matrix using the scanpy package [122] with the following parameters: n-neighbors= 10, n-pcs= 40. Additional UMAPs for each of the considered PBMC immune TFs are available in Figure S3. To create the GRN diagrams for PBMC, we used the Gephi Open Graph Viz Platform [123]. Additional GRNs for each of the PBMC immune TF families are displayed in Figure S2. PBMC heat-map dot-plots and violin-plot were created using the default scanpy parameters for these functions.

Bacillus subtilis
We used two microarray datasets for *B. subtilis*, which we label as B1 (GSE27219) and B2 (GSE67023). Both B1 and B2 underwent different normalization as part of standard microarray processing, described in detail in [116] and [117]. For the experiment "No Normalization", B1 was simply converted to integers, while B2 contained negative numbers and had to be scaled and then converted to integers so that the data represented positive integers similar to single-cell data.

To demonstrate the importance of scaling microarray data to place independently collected datasets on the same scale, we demonstrate how Min-Max Scaling improves inference in both *B. subtilis* datasets. For "Min-Max Scaling", both B1 and the positive scaled B2 dataset were subsequently normalized using the following logic. Using the observation axis, values were linearly transformed so that the minimum value was mapped to 0 and the maximum value was mapped to 1. Each value was then multiplied by 100 and converted to integers to produce the resulting expression matrix of scaled single-cell-like integers.

Inferelator, Scenic, and CellOracle Networks

Saccharomyces cerevisiae
Networks were inferred using the "multitask" workflow setting of the Inferelator for the same single-cell *S.cerevisiae* datasets described in [31]. For each algorithm, BBSR, StARS, and AMuSR, the following parameters were used: gold_standard_filter_method="keep_all_gold_standard", num_bootstraps=5. Aggregated multi-task networks were used for benchmarking, while single-task networks were disregarded for the purpose of this work. To make these networks directly comparable to PMF, we did not make use of normalization, count minimum, or meta-data options available within the Inferelator workflow.

Networks inferred with Scenic and CellOracle used the same input files, with no additional parameters specified.
