# Supplementary material for: PMF-GRN: a variational inference approach to single-cell gene regulatory network inference using probabilistic matrix factorization
Source: Genome Biol. 2024 Apr 8;25:88. doi: 10.1186/s13059-024-03226-6 (PMC11003171; doi:10.1186/s13059-024-03226-6)
Supplement: Supplementary file 5 — Additional file 5. The peer review history. [file 13059_2024_3226_MOESM5_ESM.docx]

**Review history**

**First round of review**

**Reviewer 1**

In this manuscript, a new probabilistic matrix factorization method, PMF-GRN, was represented to infer gene regulatory network from single-cell RNA sequencing data. The method requires a prior knowledge of TF-target gene interactions, which can be potentially obtained from ATAC-seq or a known network. The performance of PMF-GRN was compared with those of Inferelator, Scenic and CellOracle using two Saacharomyces cerevisiae scRNA-seq data sets and gold standard interactions as the ground truth. As a second test, PMF-GRN was also applied to two microarray data sets (after converting to single-cell-like data). Overall, I find the methodology to be interesting, and the method has a broad interest. However, additional benchmarking and testing results are required to improve the quality and robustness of the study.
1. The method is based a model of gene expression by d_n ∗ U_n V^T. Is the model sufficient to capture the gene expression distribution of a typical scRNA-seq data?
2. When analyzing the second test case, the authors used microarray data sets and converted them to integers after scaling. However, this conversion does not generate a typical scRNA-seq gene expression distribution. Effects like dropout effects were not captured in these converted data sets. The authors need to test their methods on actual scRNA-seq data, instead of data converted from an old technology. Besides, the sizes of the microarray data were not described.
3. A major concern is the ground truth and the prior network used in the benchmark. It seems that known TF-target gene interactions are used for both. The authors need to clearly state their usage in the benchmark tests. Moreover, such known interactions may be incomplete, inaccurate, and not context-specific. Thus, it may not be robust to use them as the ground truth for benchmarking. The authors need to look into the following aspects.
a. Could the authors explain why there is a drastic drop of performance when testing with the cross-validation approach? Does that mean the method is not effective in identifying new interactions?
b. How about different ratios of splits for cross-validation?
c. How about inaccurate prior network? For example, a set of partially permutated TF-target interactions to be included in the prior network.
d. It is highly recommended that the authors use simulated scRNA-seq data (e.g. using BEELINE) for benchmarking, as the ground-truth interactions are known.
4. In my view, the authors also need to demonstrate an application of the method using scRNA-seq from a mammalian system and a prior network derived from another independent data set, such as ATAC-seq. The authors have indicated such an application in the diagram in Figure 1, and mentioned in the paper (e.g., "The pipeline leverages ATAC-seq and TF binding motif data to provide binary initial guesses of gene-TF interactions."). However, no actual analysis was performed to illustrate this usage. It would also demonstrate the usefulness of the method when the ground truth and the prior network are completely independent.
5. How different are the networks (i.e., the A matrices) for individual scRNA-seq data sets versus the combined data set (related to Figure 2A)? How about using the optimal network from the first data set as the prior for the second data set?
6. There are some typos in the paper. For example, "In addition to our iid assumption over the rows of U and d, We also assume that …"

**Reviewer 2**

This paper presents a biologically-inspired matrix factorization method for gene regulatory network inference (PMF-GRN) that can extract GRNs given a prior and scRNA-Seq data. PMF-GRN shows great promise as a GRN inference tool as it is not organism specific by design, it includes hyperparameter tuning for optimal model selection, it can be easily run on large datasets as it uses GPUs, and it can be used to generate uncertainty estimates for any TF-gene interaction. The authors perform a series of experiments to show how their method compares with other state-of-the-art approaches for GRN inference, including: the Inferelator (AMuSR, BBSR, StARS variants), Scenic, and CellOracle. The proposed method was either competitive or outperformed other methods in these experiments. It was also robust against noise.
While interesting and useful, I think there are some issues that should be addressed before publication.
(i) Throughout the paper, the authors miss opportunities to provide specific examples that could motivate readers to use PMF-GRN over other tools.
In the introduction section, a message is conveyed that PMF-GRN would solve the reliability of input data, problem of new assumptions, and organism restrictions. It is not exactly clear how PMF-GRN addresses such issues compared to other tools.
Also, "these methods heavily rely on the input data used to learn the GRN, causing issues when new data becomes available or new assumptions are required in the model." (line 42). What input data? Is PMF-GRN different? (e.g. even U must be learned for each dataset).
"which makes its use much more advantageous compared to most existing methods" is vague (line 203)
The manuscript frequently contrasts the method described with the other GRN models, but without mentioning specific ones. E.g. "This feature differs from previous GRN methods, where it is often unclear which algorithm or hyperparameters to use for a given dataset."(line 350).
Perhaps a table would also be helpful in contrasting the approaches and their inputs, assumptions, methodologies, etc?
(ii) Figure 1B is confusing as Yeastract was used to create the prior. What was the role of Genome data (1B middle sub figure)? If Yeastract prior was calculated based on chromatin accessibility then having both Yeastract and genome data in 1B is misleading.
(iii) Figure 1C seems to be wrong. The figure indicates the prior network was split into 80-20 as train and validation and the hyperparameters with best performance on the validation data were selected. Later the selected model was used to infer the final GRN with full prior. Then the 20% validation data is used to test the model with the gold standard GRN. However, this seems like an overfit model is selected as an optimal model. However, in the methods section, it is mentioned that hyperparameter selection is done through creating random train-validation splits from the 80% training data. The 20% data is kept aside to evaluate the model. The figure contradicts with the description in the methods section. I think the authors should properly explain k-fold cross validation and how they are using the 80% training data to tune hyperparams and the 20% is held data during this process. I suggest using the term "test" to refer to the held out data.
(iv) It is not clear what the performance in held-out genes mean. How would PMF-GRN know the order of genes in the held out data? Is it the reason the evaluation metric performance is very low in the cross-validation experiments? If this is the case, the cross-validation experiment wouldn't mean anything. Optimal model selection would be based on random chance that a certain set of hyperparams get the order of the genes right in the held out gene set.
(v) The proposed method wasn't able to outperform Cell Oracle except in Fig 2B. What is the takeaway from Fig. 2B? Does it indicate Scenic and CellOracle are overfit models? AUPR of 0.1 is also a very low value. Detailed explanation should be provided what would be the shortcomings of Scenic, CellOracle for obtaining low AUPR here.
(vi) Baseline performance is used as a term to refer to different things in Fig. 2A (combining two expression datasets) and 2B (creating multiple folds and seeing how the performance deviates for evaluating on different folds).
(vii) The motivation for the analysis in Fig. 3, without any comparison with other methods, is not clear. Moreover, Figure 3 is labeled strangely. Why are some labels on the x axes and some in a legend at the top?
(viii) No details were provided regarding the noise added in analysis from Fig. 2C. What does 100%, .., 500% noise mean?
(ix) The datasets should be highlighted better in the methods section. The priors could also use more explanation. Was the same prior provided to all different tools? Details of how the different tools were benchmarked on the datasets would be good too.
(x) The paper didn't demonstrate dataset integration very well, except in the yeast data where the data integration aspect was glossed over. This seems like an important strength of the method.
(xi) No result present for Fig 2A: Why does CellOracle not have a "no prior"?
(xii) Typo on line 252; instead of CellOracle it should be scenic.
(xiii) "unmasking biological signal in distinct cells" is somewhat vague
(xiv) Some of this should likely be in the figure caption, not the main text: "The results for GRNs inferred without prior-known information are demonstrated by the black dots, while the gray dots demonstrate GRNs inferred with shuffled prior-known information, shown in Figure 2A" (line 287)
(xv) Seems like the paragraph starting at line 134 could simply be a sentence stating that without prior information about TF-target connectivity, the identities of the TFs will be unknown.
(xvi) Have the authors compared to IQCell?
(xvii) Since A and B are underdetermined to some degree (e.g. a change in A can be compensated for by a change in B), how confident are you that they represent the desired latent variables? How would one test these?
(xviii) It may be useful to include the lines that correspond to random performance for PR plots.
(xviii) Evaluating performance on a multicellular eukaryote dataset would help convince those working with such datasets that they should use this approach. Presumably ChIPseq or motif data could be used as priors/ground truths.
(xix) The term "well calibrated" for uncertainty estimates implies to me that the uncertainties captured in the model (posterior variances) are proportional to the probability of the interaction being correctly captured (or something similar). E.g. 25% of the interactions at a level of 25% are correct). The posterior variance . Related point: I find Fig 2D figure and caption to be quite confusing and can't really follow what's going on there. "Posterior means are cumulatively placed in bins" should this be "The TF-gene interactions are cumulatively..."

**Reviewer 3**

This paper discusses an algorithm to construct a GRN directly from single cell data called Probabilistic matrix factorization - Gene regulatory network (PMF-GRN). The method utilizes a probabilistic matrix factorization to define gene regulatory networks using a transcription factor activity (TFA) approach. The single cell expression matrix W∈R^(N*M), is factored into two submatrices U∈R^(N*K) and V∈R^(M*K) such that UV^T≈W. N represents the number of cells, M represents the number genes, and K represents the number of transcription factors (TFs). V is further decomposed into A⨀▒B, where A represents the binary present of an edge between a gene and B represents the interaction strength and direction.

The authors evaluate their performance on two single cell dataset of Saccharomyces cerevisiae and evaluated using three different tests. Across these tests PMF-GRN performs well but is comparable to other methods; in some it is outperformed by other methods. The authors use microarrays to further demonstrate their algorithm. Here also the results are mixed, where they outperform on one but not both datasets.

The overall framework has some promise, but the more rigorous experimentation and evaluation is needed to show if there is significant advantage of this framework compared to more unbiased methods that don't heavily rely on an input network. The experiments are also a bit unsatisfactory as limited set of scRNA-seq datasets are used although there are so many such datasets. More details below:

Positive aspects of the paper:

The authors implementation of a probabilistic matrix factorization algorithm for inference of a GRN from single cell data is novel.
PMF-GRN is relatively stable in the tests performed by the authors on Saccharomyces cerevisiae data, and performance is at least comparable to other methods relative in each task.
The PMF algorithm allows for the incorporation of knowledge into the inference algorithm.
Hyperparameter selection is automated for the PMF-GRN algorithm, though this relies on cross-validation which is a standard approach.
Estimations of edge confidence are provided because the model is a probabilistic framework.

Negative aspects of the paper:
There is little to no utilization of the TFA profiles. An exploration of these profiles would be an interesting addition. Perhaps a method to define active subnetworks per cell type.
This is not the first probabilistic formulation of TFA estimation. There are previous approaches which should be compared against https://genome.cshlp.org/content/early/2021/06/23/gr.265595.120.full.pdf
The model will not be able to infer new edges, especially for those TFs not in the input. This severely limits the generalization of this method to large scale GRN inference.
The size of a single cell datasets can drastically affect the quality of the inferred network. It would be nice to see the authors explore the ability of PMF to recover gold-standard networks across datasets of variable size, perhaps by downsampling. This is especially important in single cell network inference as the ability to generate large amounts of data for rare-cell types is prohibitively expensive.
The authors did not provide information on the scale of the data utilized in this study. The number of cells and genomic features in each of the S. cerevisiae samples should be provide for context. The number of samples from the two micro-array study should also be provided.
The inclusion of micro-array data in this study is poorly motivated. The method is intended for use on single cell data and thus its performance should be demonstrated on such data. The bulk microarray distribution likely has different distributions than expected in scRNA and are likely denser.
The others do not motivate the selection of prior distribution over U, V, A, and B. How these choices prior distributions affect the inference would be an interesting avenue of study. The overall model is also poorly described; it seems to make a lot of independence assumptions and it is unclear how much the probabilistic framework is actually helping.
The priors used in this study are generated are highly curated. Testing with a motif prior or ATAC-seq based prior as suggested in figure 1.B would increase the strength of the results.
Comparison to SCENIC is not fair since SCENIC does not use any prior network and relies only expression.


Minor issues:
The cell depth parameter is defined between 0-1. I do not understand the meaning of this. It seems to act as a normalization parameter but could be better explained.
In line 252, they say PMF is more robust to noise in the prior than CellOracle. This is unsupported by the data.

**Authors’ response to reviewers**

Reviewer #1: In this manuscript, a new probabilistic matrix factorization method, PMF-GRN, was represented to infer gene regulatory network from single-cell RNA sequencing data. The method requires a prior knowledge of TF-target gene interactions, which can be potentially obtained from ATAC-seq or a known network. The performance of PMF-GRN was compared with those of Inferelator, Scenic and CellOracle using two Saacharomyces cerevisiae scRNA-seq data sets and gold standard interactions as the ground truth. As a second test, PMF-GRN was also applied to two microarray data sets (after converting to single-cell-like data). Overall, I find the methodology to be interesting, and the method has a broad interest. However, additional benchmarking and testing results are required to improve the quality and robustness of the study.

1. The method is based a model of gene expression by d_n ∗ U_n V^T. Is the model sufficient to capture the gene expression distribution of a typical scRNA-seq data?

We appreciate the reviewer’s thoughtful inquiry regarding the sufficiency of our proposed model, which is based on the formulation of d_n ∗ U_n V^T to capture the gene expression distribution in scRNA-seq data. We acknowledge that the true underlying interactions governing gene expression can be highly complex and may involve non-linear relationships. However, we believe that our linear interaction model is a reasonable first-order approximation to these potentially more intricate interactions.
Several factors support our belief in the suitability of this model:
Linear models offer greater interpretability compared to complex non-linear models, making it easier to identify and understand the relationships between latent factors (U_n and V) and gene expression (W). This can provide novel insights into the biology of gene expression.
Linear models have been successfully applied in the analysis of scRNA-seq data and have provided meaningful results in which various biological systems can exhibit different behaviors.
Our experiments, as presented in this paper, show that the d_n ∗ U_n V^T model produces results that align well with the observed gene expression patterns in scRNA-seq, as validated by a gold standard. This provides empirical evidence of its adequacy for capturing the data distribution.
Linear models are computationally efficient and scalable, which is crucial for dealing with large-scale scRNA-seq datasets.
In summary, while our model may not capture the entire complexity of gene expression, we believe that it provides a sound foundation for understanding and modeling scRNA-seq data. The empirical validation provided in our study supports the use of this model as a valuable and interpretable tool for capturing gene expression distributions and further exploration of more complex interactions can be considered in future research.

2. When analyzing the second test case, the authors used microarray data sets and converted them to integers after scaling. However, this conversion does not generate a typical scRNA-seq gene expression distribution. Effects like dropout effects were not captured in these converted data sets. The authors need to test their methods on actual scRNA-seq data, instead of data converted from an old technology. Besides, the sizes of the microarray data were not described.

We appreciate the concern regarding our second test case, which involved the use of microarray data converted to integers. While we acknowledge that this conversion does not precisely replicate the characteristics of a typical single-cell RNA-seq gene expression distribution, we would like to clarify our rationale for using the B.subtilis dataset in this context.
The choice of B.subtilis was deliberate, as this organism is a well-studied model system with a reliable gold standard. Our intention in utilizing this dataset was to establish a solid foundation for our study and comprehensively investigate regulatory interactions within a reasonably well understood biological system.
Although microarray data conversion is not a direct match for scRNA-seq, the decision was made based on the robustness of the B. subtilis dataset and the extensive prior research available.
To provide further clarity on the sizes of the datasets used in our study:
Gene expression data: B1 = 266 samples x 4,218 genes, B2 = 268 samples x 3,994 genes
Prior network: 207 TFs x 2,521 genes
We would like to thank the reviewer for their thoughtful comments regarding the choice of B. subtilis. We have moved this experiment to the appendix to demonstrate an additional use-case with this dataset. In its place, we have added an additional experiment using a single cell human peripheral blood mononuclear cell (PBMC) multi-ome dataset. From this dataset, we adopted a prior constructed by masking ENCODE ChIP-seq data with the ATAC from this paired dataset. Regulatory interactions were assigned using the Inferelator-Prior package to create a prior-knowledge matrix (18,557 genes by 860 TFs). We pair this prior knowledge with a single cell gene expression matrix (11,909 cells by 36,601 genes) to infer a GRN and TFA for this cell line. In our revised manuscript, we describe our approach in detail, as well as the experiments carried out to explore the regulatory interactions and TFA inferred by PMF-GRN.

3. A major concern is the ground truth and the prior network used in the benchmark. It seems that known TF-target gene interactions are used for both. The authors need to clearly state their usage in the benchmark tests. Moreover, such known interactions may be incomplete, inaccurate, and not context-specific. Thus, it may not be robust to use them as the ground truth for benchmarking. The authors need to look into the following aspects.

We appreciate the reviewer’s attention to the sources of prior knowledge and ground truth data used in our benchmark tests. To address this concern, we would like to clarify that in our study, we utilized the YEASTRACT database as our source of prior knowledge regarding TF-target gene interactions. Furthermore, we acknowledge that the ground truth data used in our benchmark tests was curated in a separate work by Tchourine et al1. This curated gold standard dataset provides a reliable and independently validated reference for evaluating the performance of our method, as well as previously implemented works2,3,4.
We understand the importance of explicitly stating the use of these sources of information in both our text and figures to ensure transparency and clarity for readers. In response to this valuable feedback we will make it a point to provide a detailed explanation of the data usage for the yeast experiments in Figure 2.
We appreciate the reviewer’s concern regarding the potential limitation of using known interactions as the ground truth for benchmarking GRN inference methods. It is a valid point that known interactions may be incomplete, inaccurate, and not context-specific. However, we would like to emphasize that the use of known interactions as a benchmarking standard is a common practice in the field of GRN inference1,2,3,4.
In our study, we adopted the gold standard dataset curated by Tchourine et al1. as it has been widely recognized as a reference for evaluating the performance of GRN inference algorithms in yeast.
We agree that there are limitations associated with using known interactions, and we appreciate the reviewer’s concern. In our revised manuscript, we will explicitly acknowledge these limitations and discuss the context specific nature of such interactions, as well as the potential implications on the robustness of our method. We believe that this transparency will provide readers with a more comprehensive understanding of our benchmarking approach, while maintaining alignment with established practices in the GRN inference community

a. Could the authors explain why there is a drastic drop of performance when testing with the cross-validation approach? Does that mean the method is not effective in identifying new interactions?

We appreciate the reviewer’s inquiry regarding the observed drop in performance during cross-validation. The decrease in performance is an anticipated consequence of the model encountering a more limited training dataset. During this process, the model is tasked with learning from a reduced set of interactions. Additionally, when evaluated on a smaller subset of edges during testing, the model does not perform as well as when evaluated using the full gold standard. This nuanced evaluation accounts for the model’s limitations in extrapolating learned relationships to unseen data and underscores the importance of considering the trade-off between model generalization and the extent of training data during the cross-validation approach.
This decrease in performance is not unique to our method but is a common phenomenon in various GRN inference algorithms when tested in a cross-validation setting. This can be seen across all algorithms in Figure 2B.
It is worth noting that the ability to learn new interactions beyond the prior knowledge is a critical consideration in GRN inference. A major limitation in CellOracle’s method is that it cannot learn any edges that are not included in the prior knowledge. When the prior knowledge is split into training and validation sets during cross-validation, this limitation becomes particularly pronounced, as a significant portion of the edges are not included in the training data. Consequently, this significantly restricts the method’s capacity to validate and discover novel interactions during cross-validation. This underscores the importance of not only using known-interactions, but also being able to adapt and extend the model’s knowledge to accommodate new discoveries, which is a feature incorporated in our PMF-GRN approach.
In our work, we aim to strike a balance between leveraging prior-knowledge and enabling the discovery of new interactions, making it a valuable approach for GRN inference in practical applications. We appreciate the reviewer’s thoughtful consideration of these issues, and we ensure that these points are clearly addressed in our revised manuscript.

b. How about different ratios of splits for cross-validation?

We thank the reviewer for their thoughtful question regarding the cross-validation ratios in our yeast experiments. In response, we have conducted a comprehensive analysis using four different ratios (80% training - 20% validation; 60% training - 40% validation; 40% training - 60% validation; 20% training - 80% validation), with five sets of splits for each ratio. PMF-GRN consistently identifies optimal hyperparameters across all tested ratios, demonstrating its robustness. We have incorporated these findings into the revised manuscript, and have outlined these results in Figure 3B, thereby addressing this valuable suggestion and enhancing the overall rigor of our work.

c. How about inaccurate prior network? For example, a set of partially permuted TF-target interactions to be included in the prior network.

We appreciate the reviewer’s inquiry regarding the potential effects of an inaccurate prior network, especially one that contains partially permuted TF-target gene interactions. In our study, we have already considered this scenario and have addressed it in our analysis. In Figure 2C of the paper, we present a clear demonstration of the impact of adding noise to the prior network.
A description of the experiment conducted is as follows. We computed the AUPRC for networks learned with different levels of noise added to the prior. To clarify, the original prior contains 1% non-zero edges. When we refer to “100% noise” it means that we are adding 1% non-zero randomly permuted edges to introduce noise. Similarly, with “250% noise”, this corresponds to adding 2.5% noise on top of the original 1%, and “500% noise” corresponds to adding 5% noise to the prior.
This analysis clearly demonstrates how the introduction of noise impacts the performance of our and other GRN inference algorithms in the presence of an inaccurate prior network. The results provide valuable insights into the robustness and adaptability of our approach to varying levels of prior network quality. We believe that this figure effectively addresses the reviewer's concern regarding the impact of an inaccurate prior, and we have ensured that this information is appropriately highlighted and described in the revised manuscript.

d. It is highly recommended that the authors use simulated scRNA-seq data (e.g. using BEELINE) for benchmarking, as the ground-truth interactions are known.

We thank the reviewer for their insightful recommendation. We have included a section in our revised manuscript for GRN inference using the BEELINE simulated datasets.
In these experiments, we assessed the performance of PMF-GRN using the six synthetic networks of varying complexities, and found that our approach consistently outperformed the BEELINE baseline across all datasets. Additionally, we included a benchmark from the original BEELINE paper, where an AUPR ratio was computed to allow for comparison between our method and the originally tested approaches. From this benchmark, we find that PMF-GRN has competitive performance in the following synthetic datasets: linear, cycle and bifurcating converging.
These results, along with a more in depth description of the experiments conducted, can be found in the revised manuscript and in Figure 5.

4. In my view, the authors also need to demonstrate an application of the method using scRNA-seq from a mammalian system and a prior network derived from another independent data set, such as ATAC-seq. The authors have indicated such an application in the diagram in Figure 1, and mentioned in the paper (e.g., "The pipeline leverages ATAC-seq and TF binding motif data to provide binary initial guesses of gene-TF interactions."). However, no actual analysis was performed to illustrate this usage. It would also demonstrate the usefulness of the method when the ground truth and the prior network are completely independent.

We appreciate the reviewer’s insightful suggestion regarding the application of our method to scRNA-seq data from a mammalian system with a prior network derived from an independent dataset, such as ATAC-seq. In response, we have extended our analysis to include a PBMC GRN learned from a multi-omic single-cell gene expression dataset. Additionally, we have incorporated a prior-knowledge matrix derived from paired ATAC-seq data.
In performing GRN inference in a human cell line, and including an ATAC-seq derived prior, we have addressed both of these concerns, and have illustrated the applicability and utility of our method. We believe these enhancements significantly strengthen the practical relevance and robustness of our approach.

5. How different are the networks (i.e., the A matrices) for individual scRNA-seq data sets versus the combined data set (related to Figure 2A)? How about using the optimal network from the first data set as the prior for the second data set?

We appreciate the reviewer’s insightful comment regarding the comparison of the networks (A matrices) for GRNs learned on the individual scRNA-seq datasets. To address this, we computed the Intersection over Union (IoU) scores for GRNs learned by each algorithm on the two yeast datasets. The top 25% of predicted edges were retained for each GRN to compute the intersection and the union between the distinct GRNs for each method. By dividing the intersection by the union of the GRNs inferred on the individual A matrices, we quantify the similarity between the inferred GRNs.
PMF-GRN achieved the highest IoU score (15.69%) in comparison to SCENIC (3.17%), AMuSR (12.46%), BBSR (14.56%) and StARS (11.78%). CellOracle was able to achieve a higher IoU score (30.28%), however, this method is limited in its capacity to discover novel interactions beyond the prior-knowledge. This inherent constraint renders the inferred GRNs highly similar, further emphasizing PMF-GRN’s versatility in capturing diverse and comprehensive common edges.
This analysis provides an assessment of network consistency, and we have incorporated the results into our revised manuscript to address the reviewer’s inquiry effectively.

6. There are some typos in the paper. For example, "In addition to our iid assumption over the rows of U and d, We also assume that …"

We thank the reviewer for bringing our attention to these typos, we have corrected them in the revised manuscript.

Reviewer #2: This paper presents a biologically-inspired matrix factorization method for gene regulatory network inference (PMF-GRN) that can extract GRNs given a prior and scRNA-Seq data. PMF-GRN shows great promise as a GRN inference tool as it is not organism specific by design, it includes hyperparameter tuning for optimal model selection, it can be easily run on large datasets as it uses GPUs, and it can be used to generate uncertainty estimates for any TF-gene interaction. The authors perform a series of experiments to show how their method compares with other state-of-the-art approaches for GRN inference, including: the Inferelator (AMuSR, BBSR, StARS variants), Scenic, and CellOracle. The proposed method was either competitive or outperformed other methods in these experiments. It was also robust against noise.

While interesting and useful, I think there are some issues that should be addressed before publication.
(i) Throughout the paper, the authors miss opportunities to provide specific examples that could motivate readers to use PMF-GRN over other tools. In the introduction section, a message is conveyed that PMF-GRN would solve the reliability of input data, problem of new assumptions, and organism restrictions. It is not exactly clear how PMF-GRN addresses such issues compared to other tools. Also, "these methods heavily rely on the input data used to learn the GRN, causing issues when new data becomes available or new assumptions are required in the model." (line 42). What input data? Is PMF-GRN different? (e.g. even U must be learned for each dataset). "which makes its use much more advantageous compared to most existing methods" is vague (line 203)

We appreciate the reviewer’s feedback and would like to address this comment with specific examples to motivate readers to use PMF-GRN over other tools. In our paper, we aim to highlight the unique advantages and novel features of PMF-GRN in the context of GRN inference from single-cell expression and chromatin accessibility data. More specifically, we highlight the advantages of PMF-GRN in the context of other state-of-the-art scGRN inference methods, such as the Inferelator, Cell Oracle, and SCENIC.
The Inferelator is a method based on regularized regression that contains three different algorithms to choose from. a priori it is impossible to tell which algorithm will yield the best GRN in terms of AUPRC. Additionally, there is no justification for why one algorithm may perform better than the others, and in which use cases an algorithm should be selected. We refer to this issue in the text, and highlight that in comparison, PMF-GRN has a principled approach to model selection. By this we mean that there is one such model defined, where the hyperparameters for this model are selected in a principled way.
CellOracle is a method based on Bayesian ridge regression and provides competitive networks to PMF-GRN. However, this method is limited by its inability to learn edges that are not in the prior. As prior-knowledge is severely limited for almost all eukaryotes, this causes an issue when trying to infer novel regulatory edges in complex systems. In contrast, PMF-GRN can learn novel edges that are not included in the prior, as long as there is evidence of expression in the input single-cell gene expression data.
SCENIC is a method based on tree based regression, that does not achieve an AUPRC better than random. This finding is supported by previous work as well.3

To further highlight PMF-GRN:
Handling uncertainty: One of the key strengths of PMF-GRN is its ability to provide uncertainty estimates for predicted regulatory interactions. This feature is particularly valuable when dealing with limited validated interactions or incomplete gold standards. PMF-GRNs uncertainty estimates can guide users in focusing on high-confidence interactions, thus reducing the risk of experimental validation efforts on false positives
Adaptability to new data and assumptions: We can provide a hypothetical scenario where new biological data or assumptions become available. For example, our model currently focuses on modeling single-cell gene expression, however, if a new sequencing technology becomes available to capture expression, a simple adjustment of the modeling distributions and latent variables will allow us to infer a GRN without rebuilding our inference algorithm. PMF-GRNs probabilistic framework with variational inference allows seamless integration of these updates without requiring a complete algorithm redesign. This adaptability is especially important in the dynamic field of systems biology
Hyperparameter optimization: PMF-GRN uses a principled approach to hyperparameter selection through cross-validation. The final model is inferred and selected based on the log-likelihood (or its lower bound) of the data. We compare this approach to the scenario where other methods rely on heuristic model selection - meaning these methods do not perform hyperparameter search, they simply just perform inference on the data. The benefit of PMF-GRN is that it systematically explores various generative models and hyperparameter configurations to select the optimal model, which can be demonstrated through our comparative analysis.
Scalability: PMF-GRN is designed for efficiency and can perform GRN inference on a GPU, making it scalable to large single-cell gene expression datasets.
Diversely tested: PMF-GRN has been tested and demonstrated insightful results within the following organisms or datasets: S. cerevisiae, human PBMCs, and BEELINE synthetic data

The manuscript frequently contrasts the method described with the other GRN models, but without mentioning specific ones. E.g. "This feature differs from previous GRN methods, where it is often unclear which algorithm or hyperparameters to use for a given dataset."(line 350).

We thank the reviewer for pointing out this ambiguity and have updated the text to reference the Inferelator. The Inferelator contains three algorithms, of which, we cannot a priori tell which algorithm will yield the best network. We also cannot justify why one algorithm is better than another in any particular situation of GRN inference.

Perhaps a table would also be helpful in contrasting the approaches and their inputs, assumptions, methodologies, etc?

We thank the reviewer for this thoughtful suggestion. We have included a table in the supplement of our revised manuscript contrasting each GRN inference methods inputs, outputs, methodologies and pipeline.

(ii) Figure 1B is confusing as Yeastract was used to create the prior. What was the role of Genome data (1B middle sub figure)? If Yeastract prior was calculated based on chromatin accessibility then having both Yeastract and genome data in 1B is misleading.

We would like to thank the reviewer for this request of clarification. We have updated Figure 1B to demonstrate more clearly that there are two potential ways of obtaining a prior-knowledge matrix. YEASTRACT is an example of a prior-knowledge matrix obtained from a database.
To demonstrate an example of a prior-knowledge matrix obtained from chromatin accessibility, we have included this in our experiment for peripheral blood mononuclear cells (PBMCs).
We believe that these two use cases (yeast and PBMC) accurately demonstrate how genomic data as well as chromatin accessibility can be used as prior-knowledge for GRN inference.

(iii) Figure 1C seems to be wrong. The figure indicates the prior network was split into 80-20 as train and validation and the hyperparameters with best performance on the validation data were selected. Later the selected model was used to infer the final GRN with full prior. Then the 20% validation data is used to test the model with the gold standard GRN. However, this seems like an overfit model is selected as an optimal model. However, in the methods section, it is mentioned that hyperparameter selection is done through creating random train-validation splits from the 80% training data. The 20% data is kept aside to evaluate the model. The figure contradicts with the description in the methods section. I think the authors should properly explain k-fold cross validation and how they are using the 80% training data to tune hyperparams and the 20% is held data during this process. I suggest using the term "test" to refer to the held out data.

We thank the reviewer for this question and would like to clarify. The prior-knowledge was split into an 80%-20% train and validation split, where the hyperparameter search was performed in the 80% train split. Hyperparameters were selected based on the highest validation AUPRC using the 20% validation split. Following this hyperparameter search, we then used the entire prior to infer a GRN. We evaluated this GRN using an independently collected gold standard1 that was not seen by the algorithm during training. This use of data both ensures that the hyperparameters are chosen effectively, and the method is robustly tested on unseen, independent data.
In order to more clearly demonstrate our use of data, we have updated Figure 1C to reflect the independence between the prior-knowledge which is used for cross-validation based hyperparameter search, and the gold standard, which is used as a final evaluation metric.

(iv) It is not clear what the performance in held-out genes mean. How would PMF-GRN know the order of genes in the held out data? Is it the reason the evaluation metric performance is very low in the cross-validation experiments? If this is the case, the cross-validation experiment wouldn't mean anything. Optimal model selection would be based on random chance that a certain set of hyperparams get the order of the genes right in the held out gene set.

We would like to clarify that by held-out genes we mean that during our cross-validation experiments, the rows (genes) corresponding to the validation split of the prior-knowledge are zeroed out. We, however, retain the row names (genes) corresponding to these zeroed out values in order to obtain results for these genes from our matrix factorization approach.
The diminished performance in cross-validation is a consequence of inferring the GRN on a substantially reduced dataset. We opted to use the gold standard in cross-validation to emphasize the capacity of certain algorithms, like PMF-GRN, to predict edges not present in the training split. Notably, using the gold standard aids in focusing on the predictive accuracy and mitigates concerns related to potential noise in edges, with respect to the YEASTRACT prior knowledge. This deliberate choice underscores our objective of assessing algorithmic performance in predicting relevant interactions beyond the training set.

(v) The proposed method wasn't able to outperform Cell Oracle except in Fig 2B. What is the takeaway from Fig. 2B? Does it indicate Scenic and CellOracle are overfit models? AUPR of 0.1 is also a very low value. Detailed explanation should be provided what would be the shortcomings of Scenic, CellOracle for obtaining low AUPR here.

We appreciate the reviewer’s observation, and we’d like to provide a response that highlights the specific advantages of PMF-GRN in contrast to CellOracle, especially in the context of learning novel edges not included in the prior. In Figure 2A of our study, it is true that CellOracle exhibits slightly better performance than PMF-GRN. CellOracle is an excellent method for GRN inference, but is limited by its ability to learn edges that are included in the prior. This is demonstrated in Figure 2A, where CellOracle cannot learn a network without a prior, thus no black dot is present for this experiment. The method simply will not run without prior-knowledge. This is further supported by Figure 2B, where cross-validation greatly damages CellOracle’s performance, as removing the hold-out set of edges prevents CellOracle from including these edges in the final GRN.
The takeaway is that CellOracle is a good method but has other shortcomings beyond general GRN inference performance. This significantly impairs CellOracle’s ability to infer GRNs with novel interactions.
The observed low AUPR values for SCENIC in our study suggest limitations in their algorithmic design. A more detailed analysis of SCENIC should be reserved for future work to better understand the performance in terms of algorithmic design or overfitting. SCENIC involves multiple intricate steps, making it challenging to pinpoint specific areas of algorithmic failure.
Our primary focus in this study was to highlight the predictive accuracy of different algorithms in inferring GRNs on held-out genes, rather than providing an exhaustive analysis of the underlying shortcomings of each method. It is noteworthy that previous research3 has provided evidence supporting our observations, indicating that SCENIC may indeed face challenges in determining meaningful results. We appreciate the suggestion for a more in-depth exploration and will consider this for future work.

(vi) Baseline performance is used as a term to refer to different things in Fig. 2A (combining two expression datasets) and 2B (creating multiple folds and seeing how the performance deviates for evaluating on different folds).

We appreciate the reviewer’s valuable feedback regarding the use of the term “baseline performance” and understand the need for greater clarity in our manuscript. We have made the necessary revisions to only refer to baseline in the context of baseline controls (shuffled and no priors). We clarify that when the two expression datasets are combined, this is simply to test method robustness to situations where the data is not cleanly separated into “tasks”.

(vii) The motivation for the analysis in Fig. 3, without any comparison with other methods, is not clear. Moreover, Figure 3 is labeled strangely. Why are some labels on the x axes and some in a legend at the top?

We thank the reviewer for their feedback. Our motivation for the B. subtilis data was to provide an extra dataset where we had access to well established prior-knowledge / gold standard interactions.
We have decided to move this experiment (and corresponding figure) to the appendix because we have added extra datasets at the request of the reviewers.
We have clarified the figure labels to make the results more clear.

(viii) No details were provided regarding the noise added in analysis from Fig. 2C. What does 100%, .., 500% noise mean?

We thank the reviewer for their inquiry regarding the noise levels in Figure 2C. In our analysis, we refer to 100% noise, 250% noise, and 500% noise as percentages relative to the non-zero edges in the normal prior. To provide more context, the normal prior (0% added noise) contains 1% non-zero edges. Our experiment for 100% noise means that we have added another 1% of non-zero edges that are randomly permuted. Similarly for 250%, we add 2.5% non-zero randomly permuted edges, and for 500% we add 5% non-zero randomly permuted edges. We appreciate the lack of clarity as pointed out by the reviewer and have made sure to make this experiment more clear in the revised manuscript.

(ix) The datasets should be highlighted better in the methods section. The priors could also use more explanation. Was the same prior provided to all different tools? Details of how the different tools were benchmarked on the datasets would be good too.

The exact same datasets were provided to every tool to ensure stability in our experiments. We agree with the reviewer that the datasets could be better explained, and thus have included more in depth descriptions in our revised manuscript. All methods were benchmarked using exactly the same metrics for fair comparison

(x) The paper didn't demonstrate dataset integration very well, except in the yeast data where the data integration aspect was glossed over. This seems like an important strength of the method.

In the yeast experiment, it might appear that the data integration aspect was glossed over. However, we would like to clarify that in this case, the integration process was relatively straightforward. The two experiments were simply combined into one expression matrix using the pandas concat function along the row axis. This allowed us to create a dataset of 44,343 cells by 6,763 genes from the original datasets (GSE125162 = 38,225 cells by 6,763 genes and GSE144820 = 6,118 cells by 6,763 genes). We have made sure to clarify this into our revised manuscript

(xi) No result present for Fig 2A: Why does CellOracle not have a "no prior"?

CellOracle, by design, cannot learn edges that are not provided in the prior-knowledge, and thus cannot produce a network if there is no prior-knowledge.

(xii) Typo on line 252; instead of CellOracle it should be scenic.

We thank the reviewer for noticing this typo, we have corrected this in the revised manuscript.

(xiii) "unmasking biological signal in distinct cells" is somewhat vague

We agree with the reviewer that this phrase is vague. We have modified this sentence to be more precise.

(xiv) Some of this should likely be in the figure caption, not the main text: "The results for GRNs inferred without prior-known information are demonstrated by the black dots, while the gray dots demonstrate GRNs inferred with shuffled prior-known information, shown in Figure 2A" (line 287)

We agree with the reviewer that this information should be included in the caption. We have included this information in our revised caption for Figure 2.

(xv) Seems like the paragraph starting at line 134 could simply be a sentence stating that without prior information about TF-target connectivity, the identities of the TFs will be unknown.

We thank the reviewer for this suggestion, we have modified this paragraph accordingly.

(xvi) Have the authors compared to IQCell?

IQCELL is designed for reconstructing GRNs from single cell RNA-seq data in the context of developmental trajectories. Unlike conventional GRN inference methods that solely focus on network structure, IQCELL integrates pseudo-time information, capturing the temporal dynamics of gene expression during cellular development. Its unique approach involves gene selection, correction of dropout effects, and the creation of logical GRNs based on interaction hierarchy and mutual information between gene pairs. By sorting genes based on their temporal transitions and constructing a hierarchical filter, IQCELL assembles a directed gene interaction network specific to the developmental trajectory. This methodology allows IQCELL to infer executable GRNs that provide insights into the regulatory logic governing cellular differentiation. Notably, IQCELL’s emphasis on trajectory-informed network inference distinguishes it from traditional GRN methods, which do not inherently account for the temporal dimension of gene expression changes during developmental processes.
IQCELL, while an interesting and appealing method, is however not directly comparable to the methods described in this work. We believe that in future work it would be interesting to investigate the temporal dynamics of GRN inference and to use this method as a comparison for benchmarking.

(xvii) Since A and B are underdetermined to some degree (e.g. a change in A can be compensated for by a change in B), how confident are you that they represent the desired latent variables? How would one test these?

Our approach involves a binary prior information matrix (A) that encourages capturing these interactions, with its performance evaluated against gold standard interactions to ensure accurate estimation. The recovery of known interactions provides confidence in the robustness of A. On the other hand, matrix B is tasked with capturing interaction strength and directionality, representing an ongoing research focus. Interpretation of B is more challenging, and ongoing investigations aim to elucidate the directionality of interactions. Experimental validation through overexpression or knockdown experiments offers a potential avenue to explore interaction strength and direction. While our methodology provides a solid foundation for capturing binary interactions, ongoing efforts are directed towards refining the interpretation and experimental validation of matrix B to enhance the overall reliability of the inferred latent variables.

(xviii) It may be useful to include the lines that correspond to random performance for PR plots.

We appreciate the reviewers' feedback. For simplicity, we denote the performance achieved by GRNs inferred on shuffled prior knowledge, as well as no prior knowledge as gray and black dots respectively. We believe that this depiction of our results is clear and easy for visual comparison.

(xviii) Evaluating performance on a multicellular eukaryote dataset would help convince those working with such datasets that they should use this approach. Presumably ChIPseq or motif data could be used as priors/ground truths.

We thank the reviewer for their insightful feedback. We agree that evaluating our method on a multicellular eukaryote would help us to convince those working on such datasets to use our approach. For this reason, we have demonstrated our ability to infer a GRN on a single cell multi-ome peripheral blood mononuclear cell (PBMC) dataset. For the prior-knowledge used in this experiment, we opted to use a prior that was constructed by masking ENCODE derived ChIP-seq peaks with chromosomal accessibility as measured by the paired ATAC-seq.
We believe this additional experiment effectively demonstrates PMF-GRN’s success in revealing meaningful GRN predictions.

(xix) The term "well calibrated" for uncertainty estimates implies to me that the uncertainties captured in the model (posterior variances) are proportional to the probability of the interaction being correctly captured (or something similar). E.g. 25% of the interactions at a level of 25% are correct). The posterior variance . Related point: I find Fig 2D figure and caption to be quite confusing and can't really follow what's going on there. "Posterior means are cumulatively placed in bins" should this be "The TF-gene interactions are cumulatively..."

We appreciate the reviewer’s clarification on the term “well-calibrated” concerning uncertainty estimates in our model. In the context of our work, we consider the posterior variance to be well calibrated when it demonstrates an inverse correlation with the AUPRC. This correlation, as shown in Figure 2D, indicates that higher uncertainty, as captured by the posterior variance, corresponds to lower accuracy and vice versa. We acknowledge that this interpretation differs from a conventional binary classification setup, where calibration can be taken to mean that if a model is x% confident that an example has label 1, there is an x% chance that the example indeed has label 1. We have updated the figure caption to explicitly state that the analysis illustrates a clear correlation between posterior variance and aggregate accuracy. The revised caption now aims to enhance the clarity of this relationship in the context of uncertainty estimation approach.

Reviewer #3: This paper discusses an algorithm to construct a GRN directly from single cell data called Probabilistic matrix factorization - Gene regulatory network (PMF-GRN). The method utilizes a probabilistic matrix factorization to define gene regulatory networks using a transcription factor activity (TFA) approach. The single cell expression matrix W∈R^(N*M), is factored into two submatrices U∈R^(N*K) and V∈R^(M*K) such that UV^T≈W. N represents the number of cells, M represents the number genes, and K represents the number of transcription factors (TFs). V is further decomposed into A⨀B, where A represents the binary present of an edge between a gene and B represents the interaction strength and direction.

The authors evaluate their performance on two single cell dataset of Saccharomyces cerevisiae and evaluated using three different tests. Across these tests PMF-GRN performs well but is comparable to other methods; in some it is outperformed by other methods. The authors use microarrays to further demonstrate their algorithm. Here also the results are mixed, where they outperform on one but not both datasets.

The overall framework has some promise, but the more rigorous experimentation and evaluation is needed to show if there is significant advantage of this framework compared to more unbiased methods that don't heavily rely on an input network. The experiments are also a bit unsatisfactory as limited set of scRNA-seq datasets are used although there are so many such datasets. More details below:
Availability of ground truth
Common point
Positive aspects of the paper:
The authors implementation of a probabilistic matrix factorization algorithm for inference of a GRN from single cell data is novel.
PMF-GRN is relatively stable in the tests performed by the authors on Saccharomyces cerevisiae data, and performance is at least comparable to other methods relative in each task.
The PMF algorithm allows for the incorporation of knowledge into the inference algorithm.
Hyperparameter selection is automated for the PMF-GRN algorithm, though this relies on cross-validation which is a standard approach.
Estimations of edge confidence are provided because the model is a probabilistic framework.

Negative aspects of the paper:
There is little to no utilization of the TFA profiles. An exploration of these profiles would be an interesting addition. Perhaps a method to define active subnetworks per cell type.

We appreciate the reviewer’s insightful suggestion to explore the TFA profiles estimated by PMF-GRN. We would like to highlight that in order to address this concern, we have now included a section on peripheral blood mononuclear blood cells (PBMCs), where we delve into the TFA profiles in the context of 8 annotated cell types, as well as 5 distinct families of immune TFs.
Our analysis involves visualizing TFA through UMAP dimensionality reduction. UMAP allows us to observe distinct clustering of TFA profiles for each cell type. We further delve into the activity profiles of specific immune TF families such as IRF, SMAD, STAT, GATA and EGR, providing detailed visualizations of TFA profiles and exploring their regulatory interactions within the inferred GRN.
Additionally, we identify the ten most active TFs for each cell type, and present a comprehensive view of their mean TFA values and distributions. This nuanced exploration allows for a detailed understanding of TF dynamics across diverse immune cell populations with PBMCs. Moreover, we discuss the potential regulatory roles of key TFs modulating marker genes for specific cell types, offering valuable insights into the functional specialization of immune cells. Overall, our work now includes the examination of TFA profiles and also integrates them into a broader context, elucidating the regulatory networks governing PBMCs in diverse cell populations.
We would like to thank the reviewer for this suggestion, we believe the addition of TFA to our work has greatly improved the motivation and support for our PMF-GRN approach.

This is not the first probabilistic formulation of TFA estimation. There are previous approaches which should be compared against https://urldefense.proofpoint.com/v2/url?u=https-3A__genome.cshlp.org_content_early_2021_06_23_gr.265595.120.full.pdf&d=DwIGaQ&c=slrrB7dE8n7gBJbeO0g-IQ&r=xPLEVaHMZZZPlmYJbD-7IGUnRQjcl0MJ9eECvBgwAgA&m=0_XzJckjSKT1a7XafOu4B1kdCIoLUIhG3RgHa6T7qLrugaqi6V48qf-8eNGbZ-Wq&s=mnxTmZJCOJTEF6gvua09y9j1moW_GpbrsQ3UVW9He8U&e=

We appreciate the reviewer’s suggestion to compare our method with BITFAM. We have thoroughly investigated the BITFAM method and encountered several challenges that have hindered a meaningful comparison. While both methods share a probabilistic matrix factorization approach, BITFAM has distinct differences that impact its utility and performance. Notably, BITFAM constructs its prior-knowledge matrix as part of their pipeline by using the top-n most highly expressed genes, differing significantly from our methodology. We tested both their approach to prior-knowledge construction, as well as imposed our own prior during GRN inference but were unable to obtain meaningful results.
We contacted the authors to try and reconcile the reasons this method may be failing. The authors provided us with the original data used in their GitHub ReadMe, which we implemented to try and reconstruct their experiments using their code. Unfortunately, using their datasets and code, we were unable to reconstruct the demonstrated experiments. This indicates that there is some instability with this method. We further reached out to the authors of this work to see if they could reconcile any issues present in their code. Unfortunately, in the timeline of this review process, the authors of the BITFAM method have been unable to resolve the existing issues in their code, and we therefore feel we cannot include this method in our comparative analysis of GRN inference algorithms. We remain committed to ensuring a fair and rigorous evaluation of methods and ensure that we will update the manuscript should these issues be resolved in communication with the authors of BITFAM.

The model will not be able to infer new edges, especially for those TFs not in the input. This severely limits the generalization of this method to large scale GRN inference.

We appreciate the reviewer’s valuable feedback. We would like to clarify that all user-known TFs may be included in the prior-knowledge input, regardless of whether or not an edge is known a priori between this TF and its target genes. In this situation, the algorithm will determine an edge based on the expression data alone. In the case where a TF is neither expressed nor present in the prior knowledge, it will not be included in the final model.
The imposition of limited prior-knowledge can be seen as an example of a cold start problem and a zero-shot learning problem. The cold start problem is evident in cases where new TFs or target genes, not present in the initial training data, require the model to make predictions based on limited information. Similarly, the zero-shot learning challenge arises when the model is expected to infer relationships between TFs and target genes that were not explicitly part of the training set. However, we acknowledge that while this is an interesting limitation of PMF, it is somewhat beyond the scope of the current work. We aim to address and explore these challenges in future research. This comment highlights an area of great interest, and we thank the reviewer for their valuable input.

The size of a single cell datasets can drastically affect the quality of the inferred network. It would be nice to see the authors explore the ability of PMF to recover gold-standard networks across datasets of variable size, perhaps by downsampling. This is especially important in single cell network inference as the ability to generate large amounts of data for rare-cell types is prohibitively expensive.

We thank the reviewer for this valuable suggestion. We have systematically assessed PMF-GRNs performance across datasets of variable size, specifically focusing on downsampled yeast expression data. In this experiment, we employed downsampling procedures to generate datasets at sizes of 80%, 60%, 40%, and 20%, each subject to random sampling five times to ensure robustness. Notably, PMF-GRN consistently exhibited remarkable stability in AUPRC performance despite the reduction in dataset size. The sustained accuracy in inferring GRNs underscores PMF-GRNs robustness, affirming its reliability even when confronted with limited data availability. This observation holds significance, especially in the context of single cell network inference, where generating large datasets for rare cell types is often cost-prohibitive.
This experiment has been included in the paper, along with a figure demonstrating the AUPRC performance of each downsampled dataset. The method section also includes a detailed description of the organization of this experiment.

The authors did not provide information on the scale of the data utilized in this study. The number of cells and genomic features in each of the S. cerevisiae samples should be provide for context. The number of samples from the two micro-array study should also be provided.

We thank the reviewer for this observation. We have now included the dataset sizes for all datasets used in this revised manuscript.
S. cerevisiae:
GSE125162 = 38,225 cells by 6,763 genes
GSE144820 = 6,118 cells by 6,763 genes
Combined expression = 44,343 cells by 6,763 genes
YEASTRACT prior knowledge = 6,885 genes by 220 TFs
Gold standard = 993 genes by 98 TFs
Human PBMCs:
Single cell gene expression = 11,909 cells by 36,601 genes
ATAC-seq based prior knowledge = 18,557 genes by 860 TFs
B. subtilis:
GSE27219 = 266 samples by 4,218 genes
GSE67023 = 268 samples by 3,994 genes
Subtiwiki prior knowledge = 2,521 genes by 207 TFs

The inclusion of micro-array data in this study is poorly motivated. The method is intended for use on single cell data and thus its performance should be demonstrated on such data. The bulk microarray distribution likely has different distributions than expected in scRNA and are likely denser.

We thank the reviewer for their concern with the B. subtilis data. Our main motivation for using this dataset is to provide an additional example of successful GRN inference using reliable prior-knowledge and gold standard interactions. However, as this was a consistent concern among all reviewers, we have opted to include more interesting and relevant GRN inference in our main paper, and have moved this B. subtilis experiment to the appendix.

The others do not motivate the selection of prior distribution over U, V, A, and B. How these choices prior distributions affect the inference would be an interesting avenue of study. The overall model is also poorly described; it seems to make a lot of independence assumptions and it is unclear how much the probabilistic framework is actually helping.

We appreciate the reviewer’s insightful comment regarding the motivation behind the selection of prior distributions over U, V, A, and B in our model. We carefully chose these priors based on logical considerations to capture the inherent characteristics of each variable.
Firstly, U follows a log-normal distribution, considering its entries are inherently positive. The choice of a normal distribution in the log space for U is justified by the fact that TFA, being a quantity without directly measurable units, benefits from a distribution that makes minimal assumptions about the underlying data-generating process.
A is assigned a logistic-normal distribution, primarily due to the need for its range to be within the unit interval, representing the presence or absence of an interaction. The logistic distribution is suitable for binary outcomes, ensuring that A appropriately captures the binary nature of regulatory interactions.
For B, a normal distribution is chosen, as it represents strength and direction possessing an unrestricted range on the real line.
The prior over V, being the Hadamard product of A and B, is not explicitly parameterized, ensuring consistency with the dependencies between A and B.
Regarding the independence assumptions inherent in our model, these are introduced as first-order approximations tailored to the specific problem we are addressing. Although we assume independence a priori, all of the latent variables are strongly correlated a posteriori. The probabilistic framework allows us to derive uncertainty estimates for inferred interactions. The flexibility to choose prior distributions and their hyperparameters is a key strength of our approach. This flexibility enables us to incorporate a diverse range of modeling assumptions within the same framework, accommodating various degrees of confidence in prior information. The variational inference approach allows us to infer interactions robustly, irrespective of the specific choices made for the prior distributions, providing a powerful and adaptable tool for GRN inference.


The priors used in this study are generated are highly curated. Testing with a motif prior or ATAC-seq based prior as suggested in figure 1.B would increase the strength of the results.

We thank the reviewer for this insightful suggestion and agree that an ATAC-seq based prior should be demonstrated in this work. For this reason, we have performed GRN inference on a PBMC dataset, using a paired multi omic dataset, where a prior was constructed from ATAC-seq data. The details of this experiment are included in our revised manuscript.

Comparison to SCENIC is not fair since SCENIC does not use any prior network and relies only expression.

We thank the reviewer for their concern that comparison against SCENIC is not fair. We would however like to point out that SCENIC does indeed use prior-knowledge during GRN inference.5 In their work, they describe their construction of prior-knowledge through their use of CisTarget. For the purpose of our work, and for the sake of fair comparison when testing each GRN inference method, we provided the same YEASTRACT prior to all algorithms tested. SCENIC, although capable of incorporating prior-knowledge, did not manage to perform competitively with the other methods considered.

Minor issues:
The cell depth parameter is defined between 0-1. I do not understand the meaning of this. It seems to act as a normalization parameter but could be better explained.

The cell depth parameter is designed to account for variability in overall read counts obtained from a cell, attributed to experimental noise. A value of 0 implies no reads captured, while a value of 1 represents the scenario where the full set of reads is captured. This parameter provides a normalization factor to mitigate the impact of experimental variations on the interpretation of cell-wise measurements.

In line 252, they say PMF is more robust to noise in the prior than CellOracle. This is unsupported by the data.

We thank the reviewer for this observation. We have corrected this error in our revised manuscript.

References:
Tchourine, Konstantine, Christine Vogel, and Richard Bonneau. "Condition-specific modeling of biophysical parameters advances inference of regulatory networks." Cell reports 23.2 (2018): 376-388.
Jackson, Christopher A., et al. "Gene regulatory network reconstruction using single-cell RNA sequencing of barcoded genotypes in diverse environments." elife 9 (2020): e51254.
Skok Gibbs, Claudia, et al. "High-performance single-cell gene regulatory network inference at scale: the Inferelator 3.0." Bioinformatics 38.9 (2022): 2519-2528.
Tjärnberg, Andreas, et al. "Structure primed embedding on the transcription factor manifold enables transparent model architectures for gene regulatory network and latent activity inference." bioRxiv (2023): 2023-02.
Van de Sande, Bram, et al. "A scalable SCENIC workflow for single-cell gene regulatory network analysis." Nature Protocols 15.7 (2020): 2247-2276.

**Second round of review**

**Reviewer 1**

In the revision, the authors have properly addressed my questions. New examples and benchmark comparisons make the results more robust. Thus, I recommend accepting this manuscript.

**Reviewer 3**

The authors have addressed our concerns. In the response to reviewers it would have been helpful to list the specific figures, text sections that were added to address concerns.
